# Is social media important in adolescents with eating disorders?

**DOI:** 10.1192/j.eurpsy.2023.1109

**Published:** 2023-07-19

**Authors:** B. Martínez-Núñez, D. S. Cohen, R. Encinas-Encinas, A. Paniagua-Velasco, D. A. Gómez-Guimaraes, C. García-López, B. Muchada-López, M. Faya-Barrios, M. Graell-Berna

**Affiliations:** 1 Psychiatry and Clinical Psychology, Hospital Infantil Universitario Niño Jesús, Madrid, Spain

## Abstract

**Introduction:**

Eating disorders (ED) are complex entities of multicausal etiology that mainly affect adolescents and young women. For this reason, EDs frequently cause medical and psychological complications that can cause potentially irreversible developmental sequelae during adolescence.

96% of Spanish youth (15-29 years old) use daily Internet. In addition, 83% use Social Networks. Internet could be a good way to spread information through social media, websites, providing material and means to achieve the body culture purpose.

As we have seen in various papers, social media can influence and trigger the development of EDs.

**Objectives:**

The objetives of the study are to analyse the preferred social network by adolescents diagnosed with eating disorders, as well as to measure characteristic and time-use of these networks.

**Methods:**

We decided to undergo a transversal study to analyse the use of social media. For that, we developed a survey to reflect the use of the main social networks (Instagram, Facebook, Snapchat, Twitter, YouTube and Reddit) in adolescents diagnosed with eating disorders in Spain, who are in outpatient treatment in a specialised ED unit.

**Results:**

The total number of adolescents interviewed was 65; of these 96.9% were females and 3.1% males. The mean age was 14.8 years.

The preferred social network was Instagram (54%), followed by TikTok (34%) and YouTube (6%).

Most of the patients interviewed (68%) admitted checking Instagram daily, and 31% reflected spending between 1-3 hours/day. None of the adolescents reported using Facebook or Reddit.

The majority of adolescents (89%) admitted having ignored friend requests while 12% reflected the importance of having a high number of followers as a way of external validation, getting more ‘likes’ and getting to know more people.

**Conclusions:**

The obtained results reinforce the need of exploring and taking into account the use of Social Media in adolescents with ED and how it may influence their pathology. There is a need for further prospective research in this field.

**Disclosure of Interest:**

None Declared

